# Eosinophil as a biomarker for diagnosis, prediction, and prognosis evaluation of severe checkpoint inhibitor pneumonitis

**DOI:** 10.3389/fonc.2022.827199

**Published:** 2022-08-12

**Authors:** Yanlin Li, Xiaohui Jia, Yonghao Du, Ziyang Mao, Yajuan Zhang, Yuan Shen, Hong Sun, Mengjie Liu, Gang Niu, Jun Wang, Jie Hu, Min Jiao, Hui Guo

**Affiliations:** ^1^ Department of Medical Oncology, First Affiliated Hospital of Xi’an Jiaotong University, Xi’an, China; ^2^ Department of Radiology, First Affiliated Hospital of Xi’an Jiaotong University, Xi’an, China; ^3^ Department of Epidemiology and Biostatistics, School of Public Health, Xi’an Jiaotong University Health Science Center, Xi’an, China; ^4^ Department of Oncology, The First Affiliated Hospital of Shandong First Medical University and Shandong Provincial Qianfoshan Hospital, Jinan, China; ^5^ Suzhou DiYinAn Biotech Co., Ltd., Suzhou, China; ^6^ Key Laboratory of Environment and Genes Related to Diseases, Xi’an Jiaotong University, Ministry of Education of China, Xi’an, China; ^7^ Bioinspired Engineering and Biomechanics Center, Xi’an Jiaotong University, Ministry of Education of China, Xi’an, China

**Keywords:** eosinophil percentage, checkpoint inhibitor pneumonitis, immunotherapy, lung cancer, biomarker

## Abstract

**Introduction:**

Checkpoint inhibitor pneumonitis (CIP) is a common serious adverse event caused by immune checkpoint inhibitors (ICIs), and severe CIP can be life-threatening. We aimed to investigate the role of peripheral blood cells in diagnosis, prediction, and prognosis evaluation for all and severe CIP.

**Materials and methods:**

Patients with lung cancer receiving ICIs were enrolled in this retrospective study. Baseline was defined as the time of ICI initiation, endpoint was defined as the time of clinical diagnosis of CIP or the last ICI treatment, and follow-up point was defined as 1 week after CIP. Eosinophil percentages at baseline, endpoint, and follow-up point were shortened to “*E*_bas_”, “*E*_end_ and “*E*_fol_”, respectively.

**Results:**

Among 430 patients included, the incidence of CIP was 15.6%, and severe CIP was 3.7%. The *E*_end_/*E*_bas_ value was lower in patients with CIP (*p* = 0.001), especially severe CIP (*p* = 0.036). Receiver operating characteristic curves revealed that *E*_end_/*E*_bas_ could serve as a biomarker to diagnose CIP (*p* = 0.004) and severe CIP (*p* < 0.001). For severe CIP, the eosinophil percentage declined before the symptoms appeared and CT diagnosis. The eosinophil percentage significantly elevated at the follow-up point in the recovery group but not in the non-recovery group. The CIP patients with *E*_fol_/*E*_bas_ ≥1.0 had significantly prolonged overall survival (*p* = 0.024) and after-CIP survival (AS) (*p* = 0.043). The same results were found in severe CIP but without a statistical difference.

**Conclusions:**

Eosinophil percentage was associated with the diagnosis, prediction, and prognosis of CIP and severe CIP.

## Introduction

Treatment with immune checkpoint inhibitors (ICIs) has significantly improved the survival time in patients with advanced lung cancer (1–3). However, ICIs also result in immune-related adverse events (irAEs) in about 20–50%, and in some cases up to 70%, of patients (4–6), where checkpoint inhibitor pneumonitis (CIP) is one of the most common severe irAEs and the major cause of treatment-related death, especially in lung cancer (7–9). Noticeably, severe CIP needs extra attention from oncologists and respiratory physicians for its high mortality (14–35%) (10). Besides the damage caused directly by pneumonitis, severe CIP is prone to coexist with other pulmonary diseases, making its diagnosis and treatment challenging (11, 12). According to current guidelines, immunotherapy has to be discontinued permanently, significantly compromising the benefits thereof (13–15). Therefore, it is of great importance for oncologists and respiratory physicians to identify the potentially high-risk population before severe CIP occurs, to make a diagnosis in its early stages, and to identify an effective predictor for the long-term survival.

CIP is often presented with nonspecific symptoms such as fever, cough, and dyspnea (16). To diagnose CIP, other pulmonary diseases, such as lung infections and cancer progression, should be excluded first. Bacterial culture and viral nucleic acid detection in the blood and sputum could assist in infectious pneumonia diagnosis, while a sign of bronchial obstruction on X-ray or CT is valuable in the diagnosis of obstructive pneumonia. Otherwise, peripheral blood lymphocyte detection and bronchoalveolar lavage are of help for the differential diagnoses of CIP (16). However, the laboratory, radiological, and histological findings of CIP are not specific, making it challenging to distinguish CIP from other cases of pneumonia (16). Lung biopsy has been suggested but is hard to perform because of the patient’s condition. Due to the above-mentioned reasons, CIP cannot be effectively diagnosed in an early stage. It is usually only considered when the condition worsens and anti-infection or other treatments prove to be ineffective (6, 17). For severe CIP patients, early prediction is of great significance to decrease the risk of death and improve the survival time. However, repeated CT examination is not practicable considering the poor physical status of the patient and because imaging signs tend to lag behind pathological processes. Accurately predicting the prognosis in this special population is a critical challenge.

Recently, peripheral blood markers were found to be capable of serving as indicators that can be monitored continuously to predict irAEs and the outcomes of ICI treatment (18)—for instance, an increase in neutrophil and neutrophil-to-lymphocyte ratio (NLR) was found to be associated with irAEs (18). NLR, platelet-to-lymphocyte ratio (PLR), and prognostic nutrition index were considered potential predictive biomarkers for irAEs in non-small cell lung cancer (19, 20). Higher eosinophil count at baseline was found to be associated with a high risk of CIP (21). However, most researchers focused on peripheral blood markers at baseline. It should be noted that CIP is a constantly changing process, which needs dynamic monitoring. CIP, as an intermediate ending rather than a baseline status, probably shows the patients’ immune landscape changes (6, 17). Therefore, using baseline biomarkers prior to CIP to evaluate the prognosis after CIP may not be optimal. It should also be noticed that neutrophil and NLR are also elevated in a variety of inflammatory diseases, making it less effective in diagnosis in terms of specificity. Besides this, the biomarkers of CIP have rarely been tested for their efficacy in severe CIP, which is a serious challenge in immunotherapy. Examination of peripheral blood is minimally invasive and cost-effective, suggesting its potential value in clinical practice.

In this study, we conducted a retrospective analysis in patients with advanced lung cancer receiving anti-PD-1 antibody and assessed the correlation between peripheral blood cells and CIP, especially severe CIP. The value of blood cells in diagnosis, prediction, and prognosis evaluation was identified.

## Materials and methods

### Study design and patient selection

**Figure 1:**
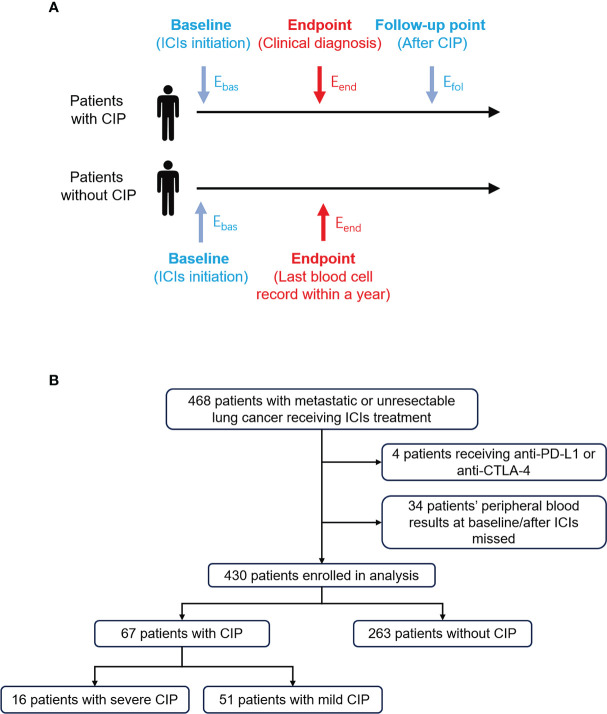
Study design and flow chart for patient selection. **(A)** Eosinophil percentage at different timepoints were extracted and defined in patients with or without CIP. The longest follow-up time for CIP was 1 year. *E*_bas_, *E*_end_, and *E*_fol_ represented eosinophil percentage at baseline, endpoint, and follow-up point, respectively. **(B)** The process of patient enrollment for analysis. CIP, checkpoint inhibitor pneumonitis; ICIs, immune checkpoint inhibitors.

For this retrospective, single-center study, patients with metastasis or unresectable lung cancer were recruited at the First Affiliated Hospital of Xi’an Jiaotong University. Patients treated with anti-PD-1 antibody combined with or without chemotherapy or anti-angiogenesis with ECOG performance status 0–2 from January 2019 to January 2021 were eligible. To exclude the marrow-suppressive effect of chemotherapy, blood cell percentage and ratio were used instead of absolute cell count. Baseline was defined as the time of ICI initiation, and endpoint was defined as the time that clinicians diagnosed CIP after considering the radiologist’s opinion. Considering the rapid change of condition and multiple intervention in the first week after CIP diagnosis, the follow-up point was defined as the first time of peripheral blood cell examination after a week of CIP diagnosis (**Figure 1A**). Only CIP occurring within 1 year from ICI initiation was considered. For patients without CIP, the time of endpoint was defined as the last time of ICI treatment within a year from ICI initiation. Patients who had received immunotherapy before or received combination therapy with anti-PD-L1 or anti-CTLA-4 antibody were excluded from our analysis.

### Diagnosis of CIP

CIP was diagnosed by one oncologist and two radiologists. The diagnosis of pneumonitis was suggested by (a) the history of ICIs use; (b) typical respiratory symptoms, such as cough, shortness of breath, decreased exercise tolerance, and exertional desaturation; and (c) typical chest CT signs, such as cryptogenic organizing pneumonia, ground glass opacities, nonspecific interstitial pneumonia, and hypersensitivity pneumonia. Meanwhile, competing diagnoses, such as lung infection and cancer progression, were also excluded (6, 17, 22). The diagnosis was double-checked by the authors during data collection. Grading of CIP was determined using the National Cancer Institute Common Terminology Criteria for Adverse Events (CTCAE) v.5.0. Delayed CIP was defined as CIP that occurred 2 months after the discontinuation of ICIs. Mild CIP was defined as grade 1 to 2 pneumonia and severe CIP as grades 3–5 pneumonia (CTCAE v.5.0). If a patient experienced CIP more than once, only the initial one diagnosed by clinicians was used in the analysis. Recovery was defined as complete absence of CIP-related symptoms, without condition worsening in CT images in 4 weeks after CIP and no recurrence.

### Data acquisition

We extracted data on the patients’ characteristics [age, sex, tumor type, number of metastases, kind of anti-PD-1 antibody, date of treatment, combination therapy type, history of smoking, radiotherapy, interstitial lung disease (ILD), and emphysema] and blood routine tests (peripheral blood cell count and percentage) from the hospital medical record system. Data on routine blood tests at the time of endpoint was acquired before steroids or other agents were used to treat the CIP. Neutrophil, lymphocyte, and eosinophil percentage was defined as the proportion of neutrophil, lymphocyte, and eosinophil in white blood cells. The NLR was calculated from absolute neutrophil counts divided by absolute lymphocyte counts, and the PLR was calculated from platelet counts divided by the absolute lymphocyte counts. Eosinophil percentages at baseline, endpoint, and follow-up point were shortened to “*E*_bas_”, “*E*_end_”, and “*E*_fol_”, respectively (**Figure 1A**). Similar abbreviations were used for other blood cell percentages and ratios.

### Statistical analysis

The eosinophil percentages (*E*_end_) and ratios (*E*_end_/*E*_bas_ and *E*_fol_/*E*_bas_) were log-transformed for statistical analyses. Log-transformed data were also used for other blood biomarker ratios and followed a normal distribution. For two-group comparisons, unpaired *t*-test, paired *t*-test, and Welch’s test were performed. Repeated-measure followed by Holm–Sidak’s *post-hoc* test was used in comparison among different times in CIP group. In the receiver operating characteristic (ROC) curve analysis, the cutoff value was determined using the highest Youden index. Univariate and multivariate analyses for risk factors were performed using binary logistic regression analysis with odd ratio and 95% confidence interval (CI). Overall survival (OS) was defined as the time interval from the initiation of ICI treatment to death or last follow-up. After-CIP survival (AS) was defined as the time interval from endpoint to death or last follow-up. The Kaplan–Meier method was used to assess the OS and AS, and the Gehan–Breslow–Wilcoxon test was applied. A comparison of clinical variables in patients dichotomized by *E*_fol_/*E*_bas_ trend was examined with chi-square test. A two-sided *α* ≥0.10 was considered to indicate homogeneity variance, and *p <*0.05 was considered statistically significant. All the analyses were carried out with the use of SPSS, version 26.0 (IBM Corp, Armonk, NY, USA) and Prism, version 9.0.0 (GraphPad Software, La Jolla, CA, USA).

## Results

### Incidence of CIP and baseline characteristics

From January 2019 to January 2021, a total of 468 patients with advanced lung cancer treated with ICIs were found (**Figure 1B**). Eventually, 430 patients were enrolled in the analysis. Among them, 67 (15.6%) were diagnosed as CIP, and 16 (3.7%) were diagnosed as severe CIP.

The baseline characteristics of the enrolled patients are summarized in **Table 1**. The median age of the patients was 62 years, and 82.3% were male patients. The major tumor types included lung adenocarcinoma (48.6%). ICI was received alone in 40 patients (9.3%) and in combination in 214 patients (90.7%). Moreover, 116 patients (27.0%) had ILD, and 120 patients (27.9%) had emphysema. Among patients with CIP, the mean age was 67 years, and 89.6% were male patients. The proportion of baseline ILD and emphysema was 58.2 and 49.3%, respectively. As for the severe CIP patients, 75.0% patients had ILD, and 50.0% patients had emphysema.

**Table 1:** Baseline characteristic of the enrolled patients.

	Total	CIP	Severe CIP
	*n* = 430 (%)	*n* = 67 (%)	*n* = 16 (%)
Age, years	62 (27–87)	63 (29–79)	64 (29–76)
Gender			
Male	354 (82.3)	60 (89.6)	13 (81.3)
Female	76 (17.7)	7 (10.4)	3 (18.8)
Cancer type			
LUAD	209 (48.6)	35 (52.2)	8 (50.0)
LUSC	165 (38.4)	25 (37.7)	5 (31.3)
SCLC	44 (10.2)	6 (9.0)	3 (18.8)
Others	12 (2.7)	1 (1.5)	0 (0.0)
Number of metastatic sites			
0	30 (7.0)	6 (9.0)	2 (12.5)
1	176 (40.9)	25 (37.3)	6 (37.5)
≥2	224 (52.1)	36 (53.7)	8 (50.0)
Treatment strategy			
Monotherapy	40 (9.3)	12 (17.9)	4 (25.0)
Combination	390 (90.7)	55 (82.1)	12 (75.0)
Baseline ILD			
Yes	116 (27.0)	39 (58.2)	12 (75.0)
No	306 (71.2)	28 (41.8)	4 (25.0)
Unknown	8 (1.9)	0 (0)	0 (0)
Baseline emphysema			
Yes	120 (27.9)	33 (49.3)	8 (50.0)
No	302 (70.2)	34 (50.7)	8 (50.0)
Unknown	8 (1.9)	0 (0)	0 (0)
Previous radiotherapy			
Yes	85 (19.8)	7 (10.4)	3 (18.8)
No	345 (80.2)	60 (89.6)	13 (81.3)
Smoking history			
Smoker	273 (63.5)	46 (68.7)	10 (62.5)
Never	155 (36.0)	21 (31.3)	6 (37.5)
Unknown	2 (0.5)	0 (0)	0 (0)
Treatment circles, median (range)	5.3 (1–23)	4.8 (1–17)	3.6 (1–9)

CIP, checkpoint inhibitor pneumonitis; LUAD, lung adenocarcinoma; LUSC, lung squamous carcinoma; SCLC, small cell lung cancer; ILD, interstitial lung disease.

### *E*_end_/*E*_bas_ assists in the diagnosis of CIP and severe CIP

First, we analyzed the association of peripheral blood biomarkers and CIP at the endpoint **(** **Supplementary Figure S1** **)** and the changes from baseline (**Supplementary Figure S2**). The ROC curve analyses revealed that the accuracy of these indexes in diagnosing CIP was close (**Supplementary Figures S3A, B**). However, for severe CIP, the *E*_end_/*E*_bas_ value was remarkably better than the others (**Supplementary Figures S3C, D**). Thus, the *E*_end_/*E*_bas_ value was chosen for further analyses.

**Figure 2:**
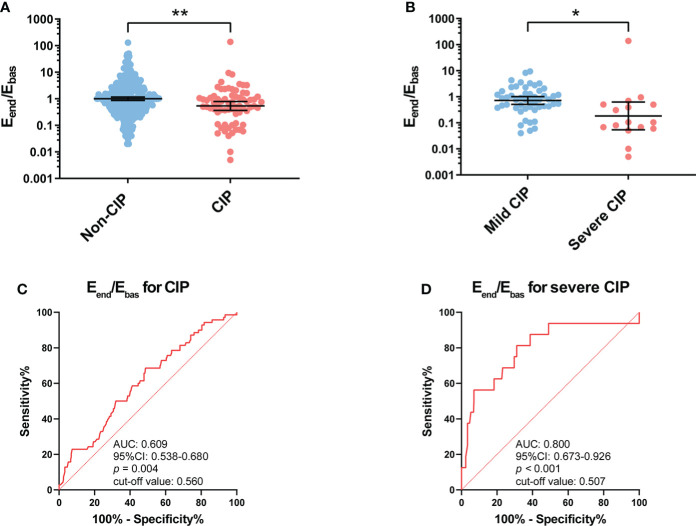
Association between *E*_end_/*E*_bas_ value and occurrence of CIP and severe CIP. **(A)** *E*_end_/*E*_bas_ value between patients with and without CIP. Bars indicate the geometric mean and 95%CI. Unpaired *t*-test. **p < 0.01. **(B)** *E*_end_/*E*_bas_ value between patients with mild and severe CIP. Bars indicate the geometric mean and 95%CI. Welch’s *t*-test. *p < 0.05. **(C)** ROC curve analysis of the sensitivity and specificity of *E*_end_/*E*_bas_ value to distinguish patients with CIP and without CIP (sensitivity, 52.2%; specificity, 68.3%; *p* = 0.004). **(D)** ROC curve analysis of the sensitivity and specificity of *E*_end_/*E*_bas_ value to distinguish patients with severe CIP and without severe CIP (sensitivity, 81.3%; specificity, 68.8%; *p* < 0.001). *E*_end_/*E*_bas_, eosinophil percentage fold change from the baseline to the endpoint; CIP, checkpoint inhibitor pneumonitis; CI, confidence interval; ROC, receiver operating characteristics.

The *E*_end_/*E*_bas_ value was significantly lower in patients with CIP compared with patients without CIP (*p* = 0.001) (**Figure 2A**). Compared with patients with mild CIP, *E*_end_/*E*_bas_ decreased more dramatically in severe CIP (*p* = 0.036) (**Figure 2B**).

The ROC curve analysis revealed that the *E*_end_/*E*_bas_ value could serve as a useful biomarker to distinguish CIP (area under the curve, AUC: 0.609, *p* = 0.004) from patients receiving anti-PD-1 antibodies (**Figure 2C**). It should also be noted that the *E*_end_/*E*_bas_ value was performed well in the diagnosis of severe CIP (AUC: 0.800, *p* < 0.001) (**Figure 2D** **).** The results of the univariable and multivariable analyses are shown in **Supplementary Table S1**. To facilitate the applicability for clinical practice, the cutoff values of *E*_end_/*E*_bas_ for CIP and severe CIP were approximately equal to 0.5. For both CIP and severe CIP, patients with *E*_end_/*E*_bas_ *<*0.5 had a significantly high risk according to the univariate and multivariate analyses.

### Eosinophil percentage reduction predicts the occurrence of severe CIP

**Figure 3:**
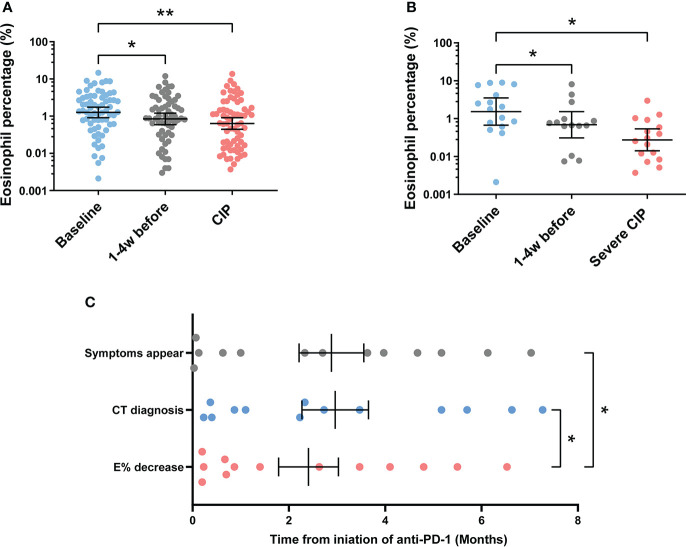
Continuous monitoring of eosinophil percentage before the endpoint. **(A)** Eosinophil percentage at different times before endpoint in patients with CIP. Bars indicate the geometric mean and 95%CI. One-way ANOVA with mixed-effects model followed by Holm–Sidak’s *post-hoc* test. **p < 0.01, *p < 0.05. **(B)** Eosinophil percentage at different times before endpoint in patients with severe CIP. Bars indicate the geometric mean and 95%CI. One-way ANOVA with mixed-effects model followed by Holm–Sidak’s *post-hoc* test. *p < 0.05. **(C)** Timeline of immune checkpoint inhibitor initiation to symptom appearance, CT diagnosis, and eosinophil percentage decrease (*E*_pre-end_ < 0.5 *E*_bas_) in patients with severe CIP. Bars indicate the mean and SEM. One-way ANOVA with mixed-effects model followed by Holm–Sidak’s *post-hoc* test. *p < 0.05. CIP, checkpoint inhibitor pneumonitis; CI, confidence interval; *E*_pre-end_, eosinophil percentage before endpoint; CT, computerized tomography.

To explore the value of eosinophil percentage in predicting the occurrence of CIP, it was monitored retrospectively for several weeks prior to the diagnosis. The results showed that the eosinophil percentage decreased 1–4 weeks in advance in patients with CIP (baseline *vs*. 1–4 weeks before, *p* = 0.034) and severe CIP (baseline *vs*. 1–4 weeks before, *p* = 0.030) (**Figures 3A,B**). To identify the decrease of eosinophil percentage in predicting the occurrence of severe CIP, we recorded the time in each severe CIP patient when the eosinophil percentage was lower than 0.5 times *E*_bas_ (half decrease time) and never increased more than that. Notably, the eosinophil percentage decreased earlier than the appearance of symptoms and CT diagnosis (*E* < 0.5 *E*_bas_: 2.4 ± 0.6 months; symptom appearance: 3.0 ± 0.7 months; CT diagnosis: 2.9 ± 0.7 months; *E* < 0.5 *E*_bas_ *vs.* symptom appearance: *p* = 0.042; *E* < 0.5 *E*_bas_ *vs.* CT diagnosis: *p* = 0.026) (**Figure 3C**).

### Eosinophil percentage elevation correlated with the recovery of CIP

**Figure 4:**
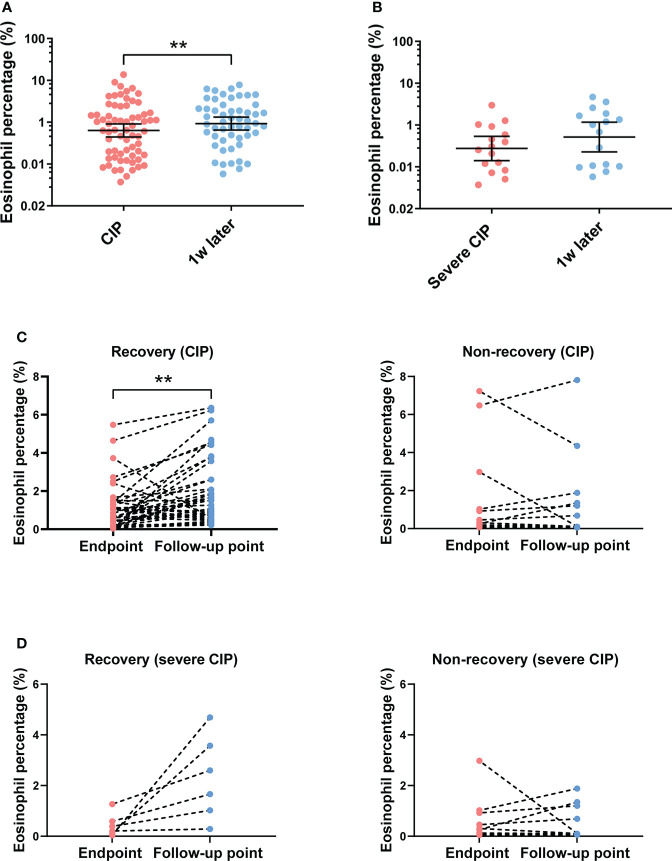
Eosinophil percentage trend and its correlation with outcomes after the endpoint. **(A)** Eosinophil percentage at different times after endpoint in patients with CIP. Bars indicate the geometric mean and 95%CI. One-way ANOVA with mixed-effects model followed by Holm–Sidak’s *post-hoc* test. **p < 0.01. **(B)** Eosinophil percentage at different times after endpoint in patients with severe CIP. Bars indicate the geometric mean and 95%CI. One-way ANOVA with mixed-effects model followed by Holm–Sidak’s *post-hoc* test. **(C)** Eosinophil percentage trend from endpoint to the follow-up point in the recovery group and the non-recovery group (patients with CIP). Paired *t*-test. **p < 0.01. **(D)** Eosinophil percentage trend from the endpoint to the follow-up point in the recovery group and the non-recovery group (patients with severe CIP). Paired *t*-test. CIP, checkpoint inhibitor pneumonitis; CI, confidence interval.

To analyze the correlation between the subsequent trend of eosinophil percentage and prognosis of CIP, the eosinophil percentage was monitored continuously for several weeks after CIP. Patients whose eosinophil percentage could not be followed after CIP were excluded (*n* = 14). For patients with CIP, the results showed that the eosinophil percentage increased after a week of the endpoint (**Figure 4A**). However, the trend was not found in patients with severe CIP (**Figure 4B**). According to the response to treatment, the patients were divided into two groups as described in “Materials and methods”: recovery group and non-recovery group. Among patients with CIP, the eosinophil percentage evaluated was significant at the follow-up point in the recovery group (*p* < 0.001), while in the non-recovery group, such change was not found (*p* = 0.597) (**Figure 4C**). Among patients with severe CIP, the same results were found but without a statistical difference (recovery group: *p* = 0.052; non-recovery group: *p* = 0.770) (**Figure 4D**)

### *E*_fol_/*E*_bas_ correlated with the long-term survival of patients with CIP and severe CIP

**Figure 5:**
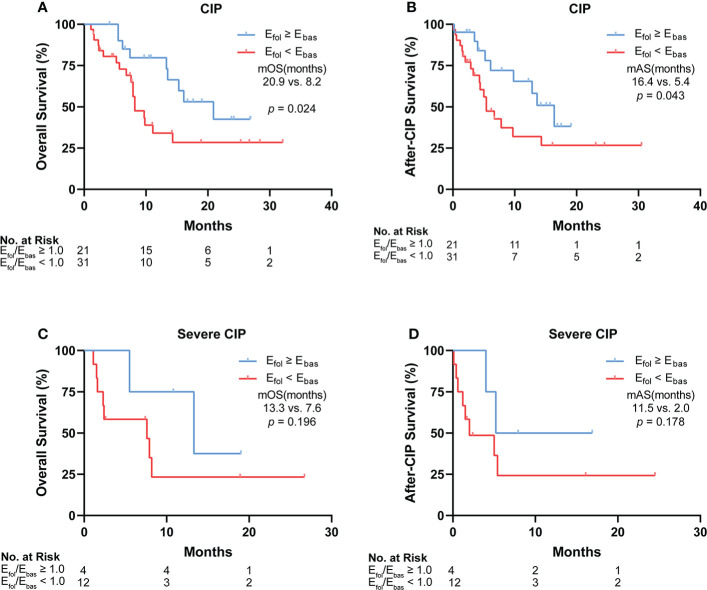
Correlation between *E*_fol_/*E*_bas_ value and the long-term survival of patients with CIP and severe CIP. **(A)** Kaplan–Meier analysis of OS in patients stratified according to *E*_fol_/*E*_bas_ value for CIP. **(B)** Kaplan–Meier analysis of after-CIP survival in patients stratified according to *E*_fol_/*E*_bas_ value for CIP. **(C)** Kaplan–Meier analysis of OS in patients stratified according to *E*_fol_/*E*_bas_ value for severe CIP. **(D)** Kaplan–Meier analysis of after-CIP survival in patients stratified according to *E*_fol_/*E*_bas_ value for severe CIP. The statistical analysis was conducted by the Gehan–Breslow–Wilcoxon test. *E*_fol_/*E*_bas_, eosinophil percentage fold change from the baseline to the follow-up point; CIP, checkpoint inhibitor pneumonitis; OS, overall survival; mOS, median overall survival; mAS, median after-CIP survival.

To analyze the impact of eosinophil percentage on survival time, Kaplan–Meier method was used. The eosinophil percentage change was compared between the follow-up point and the baseline (*E*_fol_/*E*_bas_). According to the *E*_fol_/*E*_bas_ value, we dichotomized the patients into two groups: *E*_fol_/*E*_bas_ ≥1 and *E*_fol_/*E*_bas_ *<*1. In these two groups, patient characteristics such as age, gender, cancer type, metastasis status, and steroid use exhibited no differences (**Supplementary Table S2**). Compared with the *E*_fol_/*E*_bas_ *<*1.0 group, the *E*_fol_/*E*_bas_ ≥1.0 group showed a significantly prolonged OS (median OS: 20.9 *vs.* 8.2 months, *p* = 0.024) (**Figure 5A**) and AS (median AS: 16.4 *vs.* 5.4 months, *p* = 0.043) in CIP patients (**Figure 5B**). *E*_fol_/*E*_bas_ ≥1.0 was also correlated with remarkably prolonged OS and AS in patients with severe CIP (**Figures 5C, D**). However, the differences were not statistically significant probably due to the insufficiency of people included (median OS: 13.3 *vs.* 7.6 months; *p* = 0.196; median AS: 11.5 *vs*. 2.0 months, *p* = 0.178).

## Discussion

To our knowledge, this is the first study to reveal the role of eosinophil percentage change in the diagnosis, prediction, and prognosis evaluation of CIP and severe CIP. Considering the significantly low incidence of severe CIP, although only 16 patients were enrolled, the population was still higher than most similar studies. By ROC curve analysis, we demonstrated the diagnostic value of *E*_end_/*E*_bas_ in any-grade CIP and severe CIP, which may assist in diagnosis in clinical practice. It was also observed that the eosinophil percentage was decreased several weeks before the clinical diagnosis, symptom appearance, and CT diagnosis in severe CIP. This suggested its potential value in predicting the disease. In addition, the eosinophil percentage was also related with recovery and long-term survival in CIP patients.

In our study, the *E*_end_/*E*_bas_ value has been demonstrated to have an excellent performance in detecting severe CIP, in which effective biomarkers are scarce and urgently needed. Compared with inflammatory biomarkers such as C-reactive proteins, neutrophils, and NLR, the *E*_end_/*E*_bas_ value was a unique marker to distinguish CIP from pneumonia caused by bacterial infection and cancer progression (23). The peripheral blood cell test is inexpensive and convenient. If it can help us distinguish CIP from other pneumonia or even make an early warning, it will greatly improve the accuracy of diagnosis and help avoid worse health issues. Recent studies have found that eosinophils also had strong and diverse ability in immune regulation and anti-inflammatory effect (23, 24). It has been found that eosinophils had sophisticated relationships with various cytokines and immune cells, especially T cells, which play an important role in immunotherapy response and irAEs (23, 25). Some researchers have found that eosinophils were related to the efficacy of ICI treatment, and both peripheral blood eosinophilia and eosinophil infiltration in tumor tissues have been reported (25, 26). Although the mechanism behind this correlation is still unknown, some researchers believed that it was related to the immune dysregulation caused by ICIs, and the CIP was likely to have a similar mechanism with anti-tumor immunotherapy (27). Otherwise, many people with ILD and emphysema had been enrolled in our study. However, most of the ILD and emphysema patients were asymptomatic and discovered by chest CT. No significant exacerbation of ILD or emphysema was observed except for the development of CIP.

In our study, eosinophils were abundant at baseline and decreased dramatically when CIP occurred. This trend was more remarkable in severe CIP. A week after that, the eosinophil percentage was evaluated in most patients with CIP. In other words, the reduction of eosinophil percentage occurred temporally in the course of CIP. A recent study found that eosinopenia was associated with high disease activity and autoimmunity in chronic spontaneous urticaria. As an important component of the innate immune system, eosinophils probably play a role in assisting T cells in this autoimmunity pathological process (28, 29). Considering that irAEs often mimic autoimmune diseases and the cross-antigen hypothesis, the underlying mechanism of eosinophil change in CIP may be similar to autoimmunity diseases (30, 31). During CIP, eosinophils enter into the lung tissue from the peripheral blood in response to excessive inflammation and were exhausted in the lung (32, 33). Furthermore, the intensity of eosinophil recruitment was positively correlated with CIP severity.

It should be noted that the eosinophil percentage was significantly decreased not only at the time of severe CIP but also several weeks in advance before the symptoms appeared and the CT diagnosis. The result suggested that eosinophils may enter the lungs in the early stage of CIP and cause a series of pathological progression. The cooperation of eosinophils and other cells exacerbated the inflammatory response and eventually caused symptoms and changes in the CT image (29). By continuous monitoring for eosinophil percentage, clinicians may raise awareness and discover the disease in an early stage. According to the findings, CIP should be considered when the peripheral blood eosinophil percentage decreased significantly even in the absence of special clinical manifestations and chest CT signs. The decrease of eosinophil percentage could serve as an effective biomarker to predict the development of severe CIP in clinical practice.

In addition, we also analyzed how the subsequent prognosis was affected by the eosinophil percentage change after CIP. Intriguingly, the eosinophil percentage elevation after CIP was related with recovery, especially in severe CIP. The result showed that the eosinophil percentage increased in every patient with severe CIP. In addition, patients whose *E*_fol_/*E*_bas_ *<*1.0 showed significantly poor OS and AS. The lung infiltration of eosinophils may be reduced or stopped if inflammation in the lung was controlled, and the recovery of the eosinophil percentage after treatment intervention may be a useful prognostic marker for patients undergoing CIP or severe CIP. However, the difference was not statistically significant in patients with severe CIP possibly due to the small number of participants. A previous study showed that the time to treatment failure and the survival time were longer in patients with a higher eosinophil level during ICI treatment and at baseline (21, 26, 34). The potential mechanism may be that eosinophils strengthened the antitumor response by normalizing the tumor vessels and enhancing the infiltration of CD8(+) T cells in caner tissues (35, 36). In this study, we further demonstrated the effect of high levels of eosinophil percentage after CIP on prognosis, suggesting the importance of recovering eosinophils to a high level in CIP patients. This may be because the eosinophil percentage levels reflected the development and progression of CIP. According to our results, the eosinophil percentage was correlated with outcomes in patients undergoing CIP and may serve as a potential biomarker to evaluate the patients’ prognosis. For severe CIP, the optimal timing, dose, and duration of treatment with steroids are still controversial (17). By continuously monitoring the eosinophil percentage during therapy, clinicians are able to adjust the steroid dose and the treatment pattern in time, and a more accurate prognosis assessment can be made.

There were several limitations to this study. First, this was a single-center, retrospective analysis. Second, for the low incidence of severe CIP, only 16 patients were enrolled in this group. Therefore, the role of eosinophil percentage in severe CIP needs to be confirmed in a real-world study with a larger population in the future. Third, the mechanism of eosinophils in the occurrence and development of CIP is unclear. Future work should investigate and analyze the molecular mechanism of eosinophils in severe CIP to fully understand the association between them.

## Conclusions

Our data indicate that the eosinophil percentage in peripheral blood was correlated with CIP. Continuous monitoring of eosinophil percentage trend was valuable in patients with metastatic or unresectable lung cancer receiving anti-PD-1 antibodies. The findings may be useful for personalized clinical decision-making in CIP patients, especially in severe CIP.

## Data availability statement

The raw data supporting the conclusions of this article will be made available by the authors without undue reservation.

## Ethics statement

The studies involving human participants were reviewed and approved by XJTU1AF2021LSK-001. Written informed consent for participation was not required for this study in accordance with the national legislation and the institutional requirements.

## Author contributions

Conception and design: YL, XJ, and YD. Administrative support: HS and GN. Provision of study materials or patients: MJ and HG. Collection and assembly of data: YL, XJ, ZM, and YZ. Data analysis and interpretation: YS, ML, JW, and JH. Manuscript writing: all authors. All authors contributed to the article and approved the submitted version.

## Funding

This work was supported by the National Scientific and Technological Major Special Project for Significant Creation of New Drugs (no. 2020ZX09201020), CSCO-MSD Innovation Fund (Y-MSD2020-0247), Guangdong Association of Clinical Trials/Chinese Thoracic Oncology Group, Guangdong Provincial Key Lab of Translational Medicine in Lung Cancer (no. 2017B030314120), and Clinical Key Fund of First Affiliated Hospital of Xi’an Jiaotong University (no. XJTU1AF-CRF-2019-001).

## Conflict of interest

Author Jie Hu is employed by Suzhou DiYinAn Biotech Co., Ltd.

The remaining authors declare that the research was conducted in the absence of any commercial or financial relationships that could be construed as a potential conflict of interest.

## Publisher’s note

All claims expressed in this article are solely those of the authors and do not necessarily represent those of their affiliated organizations, or those of the publisher, the editors and the reviewers. Any product that may be evaluated in this article, or claim that may be made by its manufacturer, is not guaranteed or endorsed by the publisher.
