# Supplementary material for: Eosinophil as a biomarker for diagnosis, prediction, and prognosis evaluation of severe checkpoint inhibitor pneumonitis
Source: Front Oncol. 2022 Aug 12;12:827199. doi: 10.3389/fonc.2022.827199 (PMC9413068; doi:10.3389/fonc.2022.827199)
Supplement: Supplementary file 1 [file DataSheet_1.docx]

**Legend for supplementary figures**

**Supplemental Fig. 1** Association between peripheral blood markers and CIP at the time of CIP occurrence. **A** Baseline neutrophil percentage between patients with CIP and without CIP. Bars indicate the mean and SEM. Unpaired t test. ****p* < 0.001. **B** Baseline neutrophil percentage among different severity of CIP. Bars indicate the mean and SEM. Unpaired t test. The same analysis was conducted in lymphocyte percentage (**C**, **D**), NLR (**E**, **F**), eosinophil percentage (**G**, **H**) and PLR (**I**, **J**). Bars indicate the mean and SEM or geometric mean and 95%CI. Statistical analysis included unpaired t test (**A, B D, E, F, J**) and Welch’s t test (**C, G, H, I)**. **p* < 0.05, ***p* < 0.01, ****p* < 0.001.CIP, checkpoint inhibitor pneumonitis; NLR, Neutrophil lymphocyte ratio; PLR, platelet lymphocyte ratio; CI, confidence interval.

**Supplemental Fig. 2** Peripheral blood markers change from baseline to the occurrence of CIP. **A** Neutrophil percentage change (N_end_/N_bas_) between patients with CIP and without CIP. Bars indicate the geometric mean and 95%CI. Unpaired t test. **p* < 0.05. **B** Neutrophil percentage change (N_end_/N_bas_) among different severity of CIP. Bars indicate the geometric mean and 95%CI. Unpaired t test. The same analysis was conducted in lymphocyte percentage (L_end_/L_bas_) **(C, D)**, NLR (NLR_end_/NLR_bas_) **(E, F)** and PLR (PLR_end_/PLR_bas_) **(G, H)**. Bars indicate the geometric mean and 95%CI. Statistical analysis included Welch’s t test **(A, B, E F)** and unpaired t test **(C, D, G H)**. The results for eosinophil percentage (E_end_/E_bas_) were shown in Fig. 2. **p* < 0.05. NLR, neutrophil lymphocyte ratio; PLR, platelet lymphocyte ratio; CI, confidence interval.

**Supplemental Fig. 3** ROC curve analysis of peripheral blood markers at the time of CIP occurrence and the changes. **A** ROC curve analysis of the sensitivity and specificity of neutrophil percentage (N_end_/N_bas_), lymphocyte percentage (L_end_/L_bas_) NLR (NLR_end_ /NLR_bas_), eosinophil percentage (E_end_/E_bas_) and PLR (PLR_end_/PLR_bas_) change from baseline to the endpoint to distinguish patients with CIP and without CIP. **B** ROC curve analysis of the sensitivity and specificity of neutrophil percentage (N_end_), lymphocyte percentage (L_end_), NLR (NLR_end_), eosinophil percentage (E_end_) and PLR (PLR_end_) at the time of CIP occurrence to distinguish patients with CIP and without CIP. **C** ROC curve analysis of the sensitivity and specificity of neutrophil percentage (N_end_/N_bas_), lymphocyte percentage (L_end_/L_bas_) NLR (NLR_end_ /NLR_bas_), eosinophil percentage (E_end_/E_bas_) and PLR (PLR_end_/PLR_bas_) change from baseline to the endpoint to distinguish patients with severe CIP and without severe CIP. **D** ROC curve analysis of the sensitivity and specificity of neutrophil percentage (N_end_), lymphocyte percentage (L_end_), NLR (NLR_end_), eosinophil percentage (E_end_) and PLR (PLR_end_) at the time of CIP occurrence to distinguish patients with severe CIP and without severe CIP. ROC, receiver operating characteristics; CIP, checkpoint inhibitor pneumonitis; NLR, Neutrophil lymphocyte ratio; PLR, platelet lymphocyte ratio.
