# Supplementary figures and images for: Eosinophil as a biomarker for diagnosis, prediction, and prognosis evaluation of severe checkpoint inhibitor pneumonitis
Source: Front Oncol. 2022 Aug 12;12:827199. doi: 10.3389/fonc.2022.827199 (PMC9413068; doi:10.3389/fonc.2022.827199)

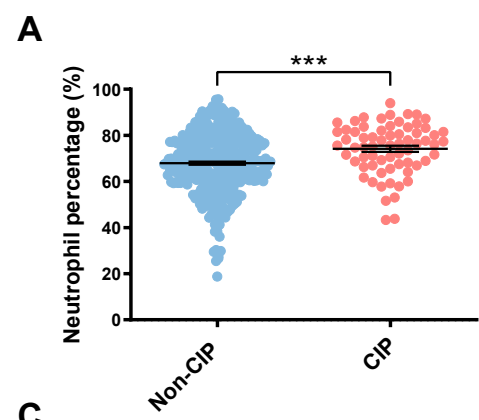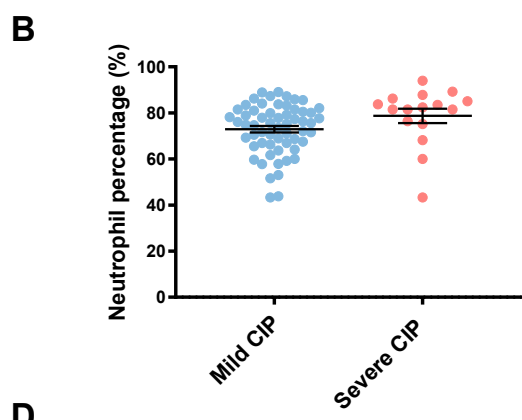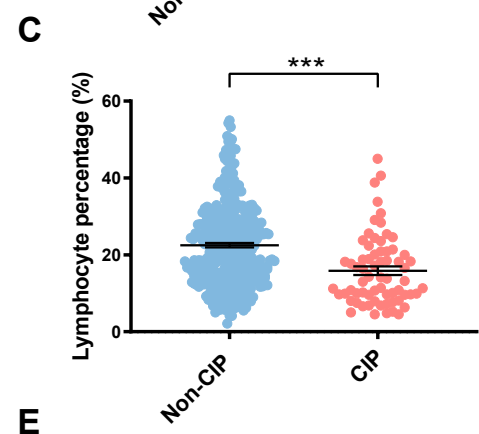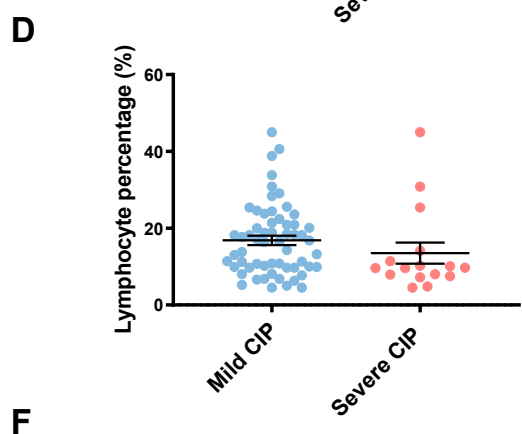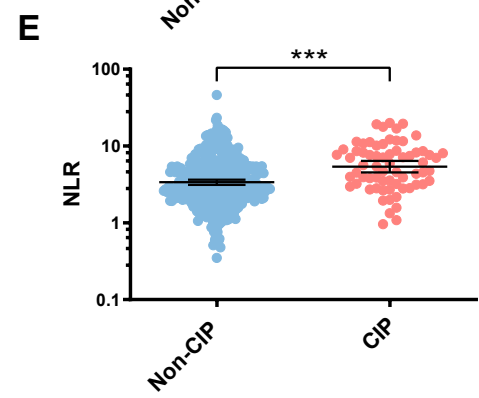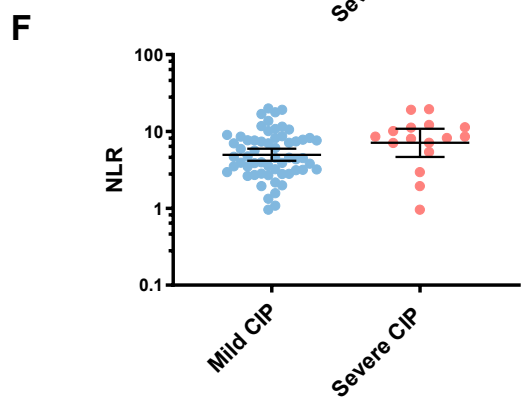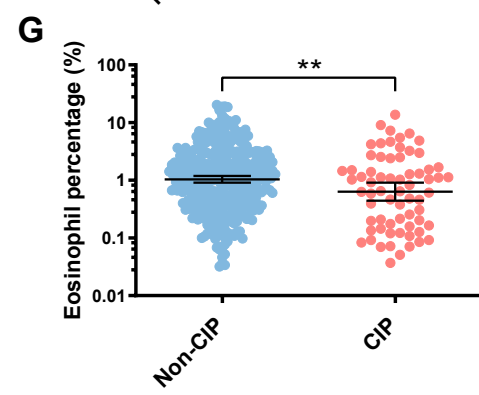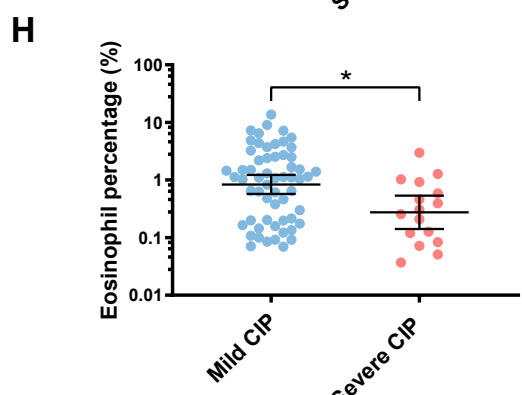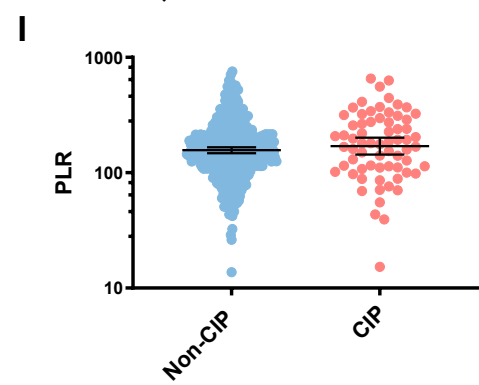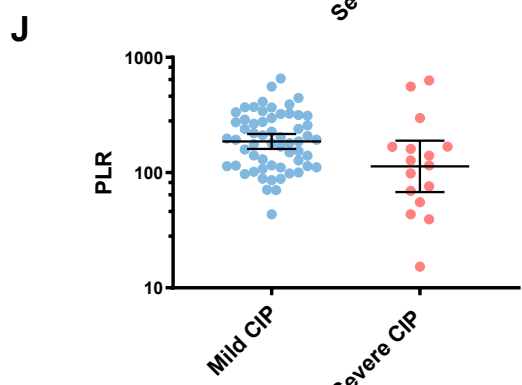

Supplement: Supplementary file 2 [file Image_1.pdf]

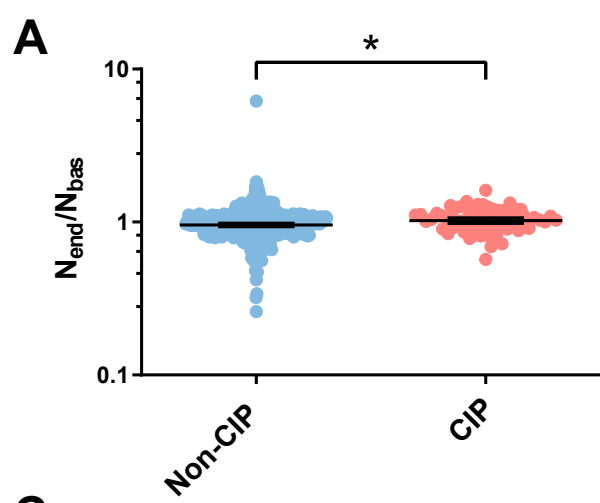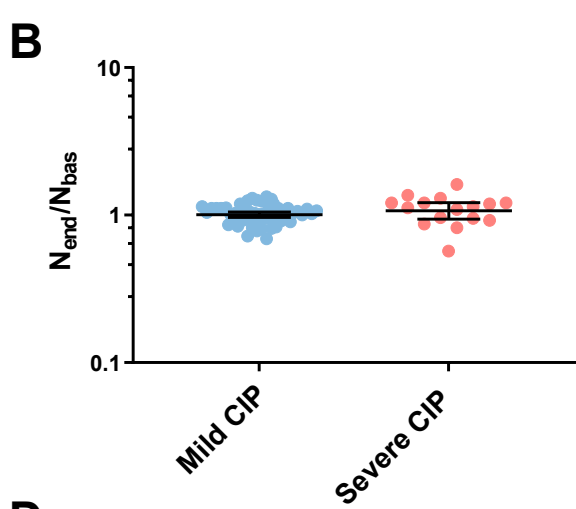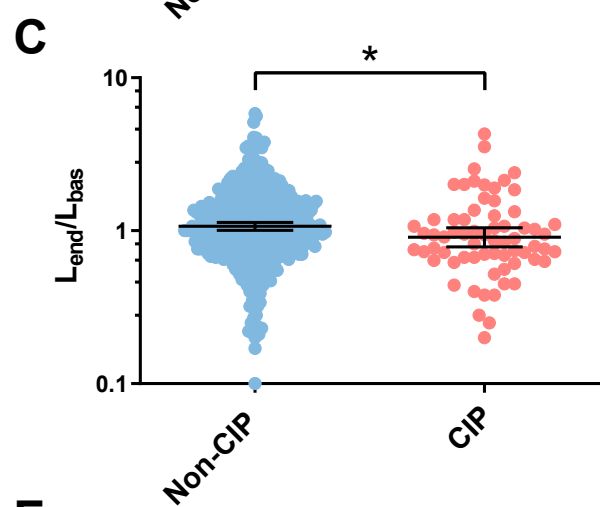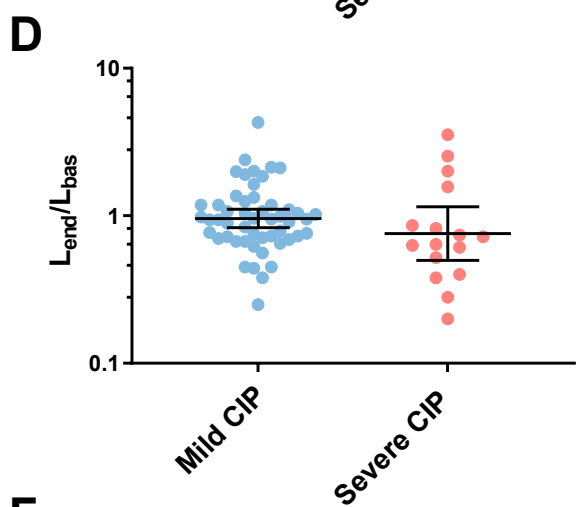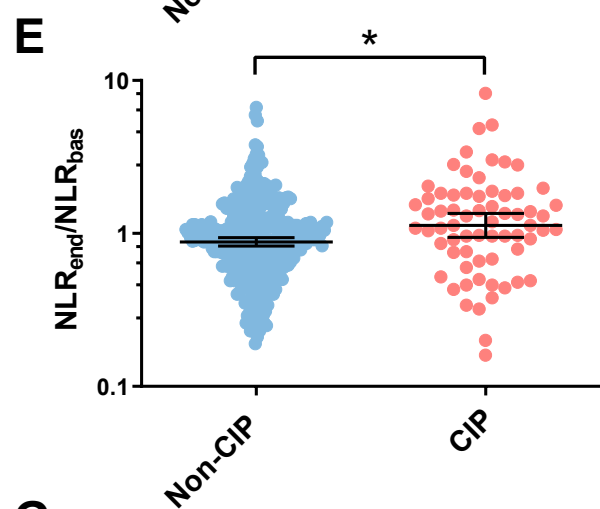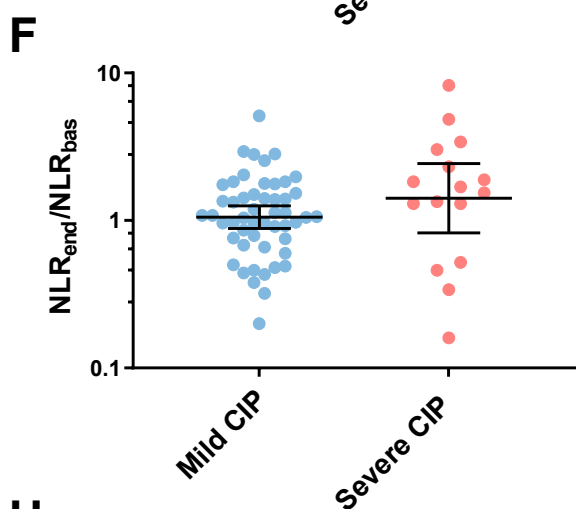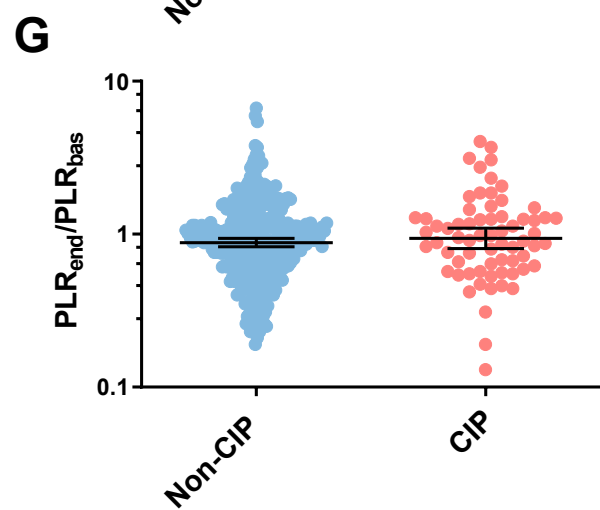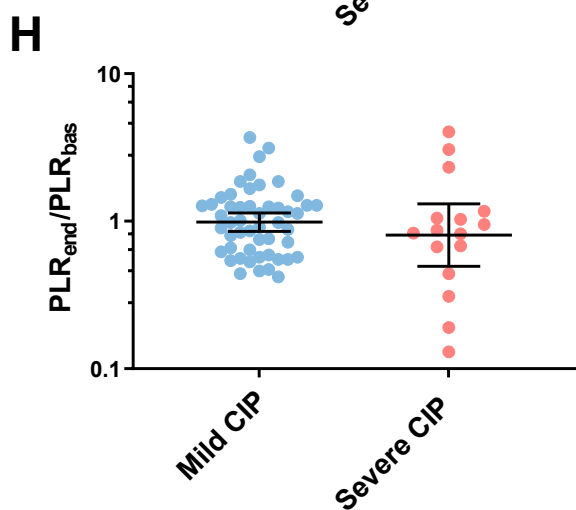

Supplement: Supplementary file 3 [file Image_2.pdf]

**A**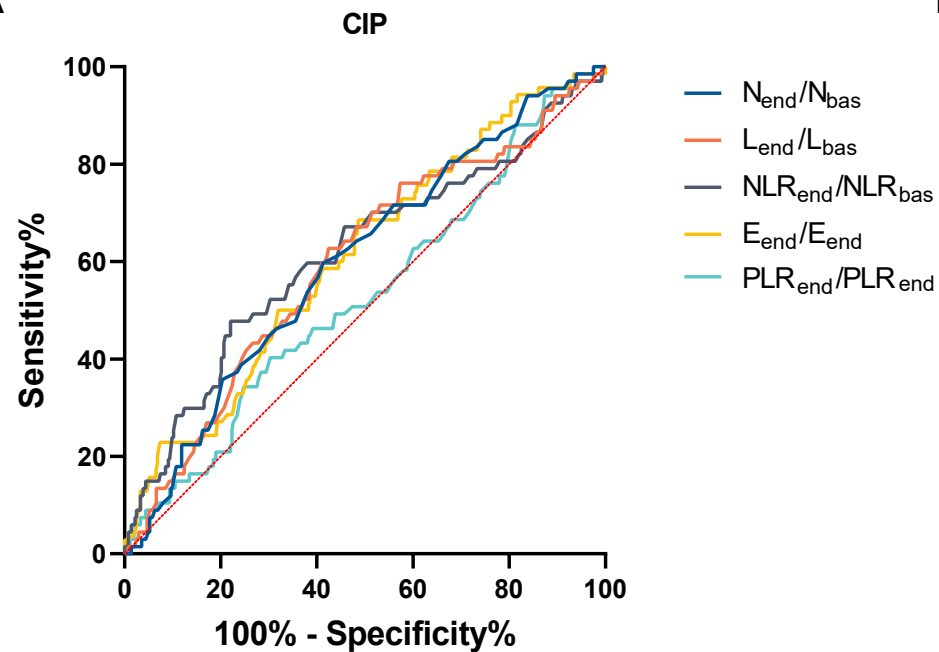**B**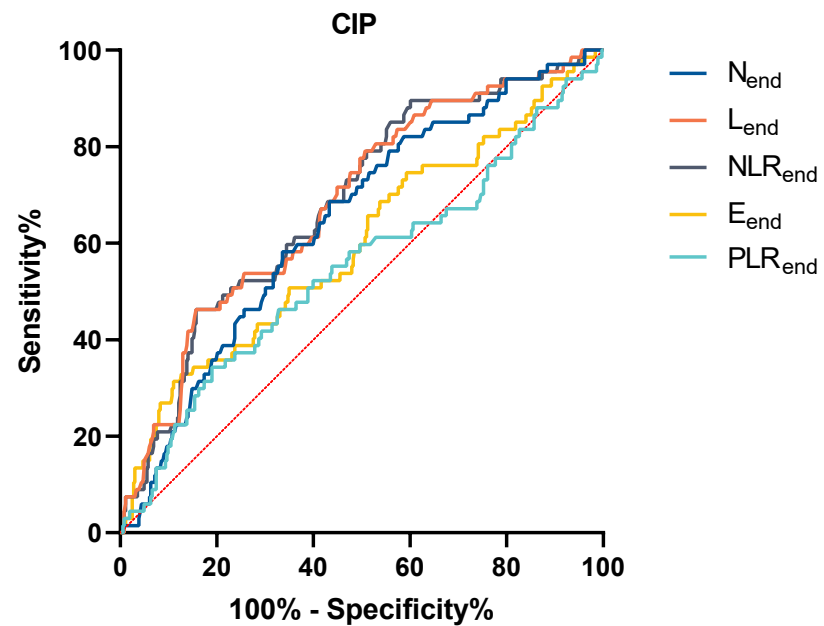**C**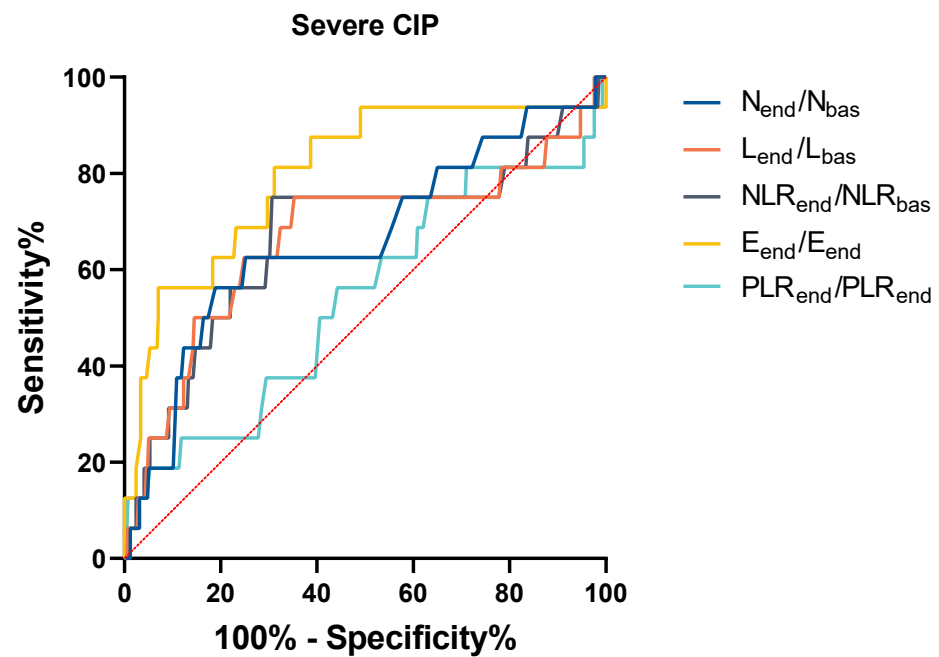**D**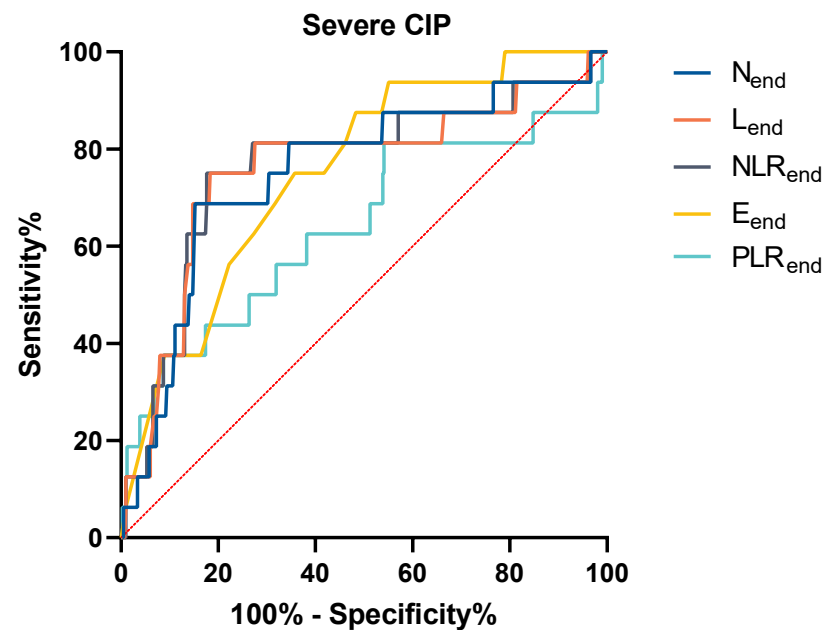

Supplement: Supplementary file 4 [file Image_3.pdf]
