# Supplementary material for: Eosinophil as a biomarker for diagnosis, prediction, and prognosis evaluation of severe checkpoint inhibitor pneumonitis
Source: Front Oncol. 2022 Aug 12;12:827199. doi: 10.3389/fonc.2022.827199 (PMC9413068; doi:10.3389/fonc.2022.827199)
Supplement: Supplementary file 5 [file Table_1.docx]

|  | **Supplementary Table 1** Logistic regression analysis for the risk factors of CIP and severe CIP | | | | | | | | | |
| --- | --- | --- | --- | --- | --- | --- | --- | --- | --- | --- |
|  | | CIP | | | |  | Severe CIP | | | |
|  | | Univariate Analysis | | Multivariate Analysis | |  | Univariate Analysis | | Multivariate Analysis | |
|  |  | OR (95% CI) | *p* value | OR (95% CI) | *p* value |  | OR (95% CI) | *p* value | OR (95% CI) | *p* value |
| Age  (≥ 65 vs.< 65) | | 2.1 (1.2-3.5) | **0.007** | 1.5 (0.8-2.7) | 0.162 |  | 2.6 (0.9-7.2) | 0.073 | 1.7 (0.6-5.2) | 0.356 |
| Gender  (male vs. female) | | 2.0 (0.9-4.6) | 0.097 | 1.3 (0.5-3.1) | 0.569 |  |  |  |  |  |
| Previous RT  (yes vs. no) | | 0.4(0.2-1.0) | **0.042** | 0.5 (0.2-1.2) | 0.110 |  |  |  |  |  |
| IPD  (yes vs. no) | | 4.7 (2.7-8.1) | **< 0.001** | 3.6 (2.0-6.5) | **< 0.001** |  | 8.3 (2.6-26.2) | **< 0.001** | 7.0 (2.1-24.1) | **0.002** |
| Emphysema  (yes vs. no) | | 3.2 (1.9-5.4) | **< 0.001** | 1.9 (1.0-3.5) | **0.047** |  | 2.8 (1.0-7.5) | **0.048** | 1.4 (0.5-4.3) | 0.557 |
| E_end_/E_bas_  (≥ 0.5 vs. < 0.5) | | 2.0 (1.2-3.4) | **0.011** | 2.1 (1.2-3.7) | **0.011** |  | 6.6 (2.1-20.9) | **0.001** | 6.8 (2.1-22.1) | **0.001** |
| *Age (*≥ *65 or <65), gender, baseline IPD, emphysema, E_end_/E_bas_ (≥ 0.5 vs. < 0.5), previous RT, smoking history and treatment strategy (monotherapy or combination) were analyzed in univariate analysis. Variables with p value < 0.10 in univariate models (CIP: age, gender, previous RT, baseline IPD, emphysema and E_end_/E_bas_; severe CIP: age, baseline IPD, emphysema and E_end_/E_bas_ ) were analyzed in multivariate analysis model. Only* *variables analyzed in multivariate analysis were shown. CIP, checkpoint inhibitor pneumonitis; OR, odds ratio; CI, confidence interval; RT, radiotherapy; IPD, interstitial pulmonary disease; E_end_, eosinophil percentage at the endpoint; E_bas_, eosinophil percentage at the baseline;* | | | | | | | | | | |
