# Supplementary material for: Eosinophil as a biomarker for diagnosis, prediction, and prognosis evaluation of severe checkpoint inhibitor pneumonitis
Source: Front Oncol. 2022 Aug 12;12:827199. doi: 10.3389/fonc.2022.827199 (PMC9413068; doi:10.3389/fonc.2022.827199)
Supplement: Supplementary file 6 [file Table_2.docx]

| **Supplementary Table 2** Comparison of clinical variables in CIP patients dichotomized via E_fol_/E_bas_. | | | |
| --- | --- | --- | --- |
|  | E_fol_/E_bas_ ≥ 1 | E_fol_/E_bas_ < 1 | *p* value |
|  | n=20 (%) | n=33 (%) |  |
| Age (years) |  |  |  |
| ≥ 65 | 11 (55.0) | 17 (51.5) | 0.805 |
| < 65 | 9 (45.0) | 16 (48.5) |  |
| Gender | | | |
| Male | 19 (95.0) | 28 (84.8) | 0.494 |
| Female | 1 (5.0) | 5 (15.2) |  |
| Cancer type | | | |
| NSCLC | 16 (80.0) | 31 (93.9) | 0.269 |
| SCLC | 4 (20.0) | 2 (6.1) |  |
| Metastasis | | | |
| Yes | 13 (65.0) | 22 (66.7) | 0.901 |
| No | 7 (35.0) | 11 (33.3) |  |
| Treatment strategy | | | |
| Monotherapy | 1 (5.0) | 6 (18.1) | 0.339 |
| Combination | 19 (95.0) | 27 (81.8) |  |
| CIP grade |  |  |  |
| Severe | 4 (20.0) | 12 (36.4) | 0.208 |
| Mild | 16 (80.0) | 21 (63.6) |  |
| Treatment line |  |  |  |
| First line | 12 (60.0) | 19 (57.6) | 0.862 |
| Sec or more | 8 (40.0) | 14 (42.4) |  |
| Treatment for CIP |  |  |  |
| Steroid | 5 (25.0) | 14 (42.4) | 0.200 |
| Non-steroid | 15 (75.0) | 19 (57.6) |  |
| *CIP, checkpoint inhibitor pneumonitis;* *E_fol_/E_bas_,* *eosinophil percentage fold change from the baseline to the follow-up point; NSCLC, non-small cell lung cancer; SCLC, small cell lung cancer; Sec or more, second line or more.* | | | |
